# Jasmonate-independent regulation of digestive enzyme activity in the carnivorous butterwort *Pinguicula* × *Tina*

**DOI:** 10.1093/jxb/eraa159

**Published:** 2020-03-27

**Authors:** Ondřej Kocáb, Jana Jakšová, Ondřej Novák, Ivan Petřík, René Lenobel, Ivo Chamrád, Andrej Pavlovič

**Affiliations:** 1 Department of Biophysics, Centre of the Region Haná for Biotechnological and Agricultural Research, Faculty of Science, Palacký University, Šlechtitelů 27, Olomouc, Czech Republic; 2 Laboratory of Growth Regulators, Institute of Experimental Botany, The Czech Academy of Sciences and Faculty of Science, Palacký University, Šlechtitelů 27, Olomouc , Czech Republic; 3 Department of Protein Biochemistry and Proteomics, Centre of the Region Haná for Biotechnological and Agricultural Research, Faculty of Science, Palacký University, Šlechtitelů 27, Olomouc, Czech Republic; 4 Max Planck Institute of Molecular Plant Physiology, Germany

**Keywords:** Butterwort, carnivorous plant, digestive enzymes, electrical signals, jasmonic acid, *Pinguicula*, protease, variation potential

## Abstract

Carnivorous plants within the order Caryophyllales use jasmonates, a class of phytohormone, in the regulation of digestive enzyme activities. We used the carnivorous butterwort *Pinguicula* × *Tina* from the order Lamiales to investigate whether jasmonate signaling is a universal and ubiquitous signaling pathway that exists outside the order Caryophyllales. We measured the electrical signals, enzyme activities, and phytohormone tissue levels in response to prey capture. Mass spectrometry was used to identify proteins in the digestive secretion. We identified eight enzymes in the digestive secretion, many of which were previously found in other genera of carnivorous plants. Among them, alpha-amylase is unique in carnivorous plants. Enzymatic activities increased in response to prey capture; however, the tissue content of jasmonic acid and its isoleucine conjugate remained rather low in contrast to the jasmonate response to wounding. Enzyme activities did not increase in response to the exogenous application of jasmonic acid or coronatine. Whereas similar digestive enzymes were co-opted from plant defense mechanisms among carnivorous plants, the mode of their regulation differs. The butterwort has not co-opted jasmonate signaling for the induction of enzyme activities in response to prey capture. Moreover, the presence of alpha-amylase in digestive fluid of *P.* × *Tina*, which has not been found in other genera of carnivorous plants, might indicate that non-defense-related genes have also been co-opted for carnivory.

## Teaser

Some carnivorous plants co-opted jasmonate signaling for prey capture from plant defense mechanisms. However, the carnivorous genus butterwort (*Pinguicula*) does not use jasmonates for the regulation of enzyme activities.

## Introduction

The carnivorous plants have evolved specialized leaves or leaf parts that function as traps for prey capture and digestion to obtain scarce nutrients. This adaptation to low nutrient content in the soil has independently evolved by convergent evolution at least 10 times in several orders of flowering plants (Albert *et al.*, 1992; Givnish *et al.*, 2015; Fleischmann *et al.*, 2018). Whereas the mechanisms of prey capture have been studied in great detail during the past two centuries, the process of digestion was almost completely unknown. The first endogenous enzyme in carnivorous plants was described only at the beginning of this century in the pitcher plant (genus *Nepenthes*), which resolved the longstanding question of whether prey digestion is mediated by symbiotic microorganisms or plant-derived enzymes (Athauda *et al.*, 2004). In the decade that followed, the development of mass spectrometry techniques enabled the discovery of over 20 other digestive enzymes in different species of carnivorous plants, which are surprisingly very similar across distantly related taxa (Eilenberg *et al.*, 2006; Hatano and Hamada, 2008; 2012; Rottloff et al., 2011; 2016; Lee *et al.*, 2016; Fukushima *et al.*, 2017; Krausko *et al.*, 2017). Yet, the mechanism by which the secretion of these digestive enzymes is regulated by stimuli from prey remained unknown until Escalanté-Pérez *et al*. (2011) found that a phytohormone from the group of jasmonates, 12-oxo-phytodienoic acid (OPDA), was responsible for activation of the digestive process in Venus flytrap (*Dionaea muscipula*). An increased level of the true bioactive compound in jasmonate signaling, the isoleucine conjugate of jasmonic acid (JA-Ile), was later found in Venus flytrap, sundew plant (*Drosera capensis*), and the pitcher plant *Nepenthes alata* in response to prey capture (Nakamura *et al.*, 2013; Libiaková *et al.*, 2014; Yilamujiang *et al.*, 2016; Krausko *et al.*, 2017; Pavlovič *et al.*, 2017). The binding of JA-Ile to CORONATINE INSENSITIVE1 (COI1) protein as part of a co-receptor complex mediates the ubiquitin-dependent degradation of JASMONATE ZIM-DOMAIN (JAZ) repressors, resulting in the activation of jasmonate-dependent gene expression (Chini *et al.*, 2007; Thines *et al.*, 2007; Fonseca *et al.*, 2009; Sheard *et al.*, 2010). In ordinary plants, JA-Ile is responsible for the activation of defense mechanisms after herbivore attack or wounding, and it was postulated that the carnivorous plants co-opted the jasmonate signaling pathway for prey capture (Pavlovič and Saganová 2015; Bemm *et al.*, 2016; Pavlovič and Mithöfer, 2019). Unfortunately, the studies to date have generally been confined to three genera of carnivorous plants (*Drosera*, *Dionaea*, and *Nepenthes*), which all are within the order Caryophyllales (or, according to some authors, the separate order Nepenthales; Fleischmann *et al.*, 2018) and are monophyletic. Therefore, it remains unclear whether the jasmonate signaling pathway is a universal and ubiquitous signaling pathway in other phylogenetic lineages of carnivorous plants.

In this study, we focused on carnivorous plants of the genus *Pinguicula* (butterworts), which belongs to the order Lamiales and is distantly related to the Venus flytrap, sundew, and pitcher plant (Albert *et al.*, 1992; Givnish, 2015). Most species of *Pinguicula* have a basal rosette of compact leaves that are more or less broadly ovate, and only a few species (*Pinguicula heterophylla*, *Pinguicula gypsicola*) have filiform upright leaves. Some species can bend their leaf edges slightly in response to prey capture, while others have no such ability (Fleischmann and Roccia, 2018). The leaves are covered by two types of glands. The stalked glands produce sticky mucilage and serve mainly for prey capture, but with the capacity to produce their own digestive enzymes. The sessile glands are the main site for the production of digestive enzymes (Heslop-Harrison and Knox, 1971; Heslop-Harrison and Heslop-Harrison, 1980, 1981; Legendre, 2000; Heslop-Harrison, 2004). Both types of digestive glands of *Pinguicula* share a special characteristic with the Venus flytrap and sundew in that they do not secrete enzymes until stimulated by the presence of prey (Darwin, 1875). It has been postulated that the glands of *Pinguicula* undergo a type of total autophagy and are simply a sac of enzymes that are discharged in response to prey capture, in contrast to the jasmonate-mediated expression/secretion of digestive enzymes in the carnivorous plants within the order Caryophyllales. The initial event associated with the onset of secretion is the rapid movement of chloride ions followed by water across the glands, flushing out the stored enzymes from the cell walls of the glands (Heslop-Harrison and Knox, 1971; Heslop-Harrison and Heslop-Harrison, 1980). Contrary to this eccrine hypothesis, Vassilyev and Muravnik (1988a , b) showed that the glands remain highly active during the entire secretion process and additional digestive enzymes are synthesized and secreted into the digestive fluid after stimulation, much in common with the process in Venus flytrap. In this study, we aimed to shed light on this discrepancy in the view of jasmonate signaling within a less-studied genus of carnivorous plant, *Pinguicula*. We were interested in whether the jasmonate signaling pathway was co-opted for plant carnivory outside the order Caryophyllales. We measured electrical activity, analyzed the composition of the digestive fluid and its enzymatic activity in response to prey capture, and assessed endogenous phytohormone content. We did not find any evidence that butterworts use jasmonate signaling for the induction of enzyme activities.

## Materials and methods

### Plant material and experimental setup

**Fig. 1.**
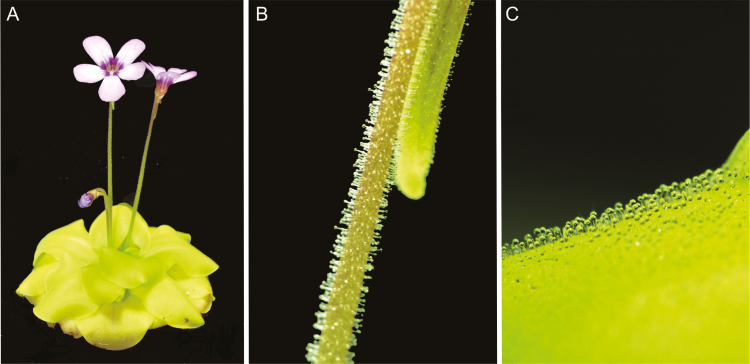
Butterwort *Pinguicula* × *Tina*. (A) Whole plant. (B) Flower stalk covered with digestive glands. (C) Leaf covered with stalked and sessile glands.

We used a horticultural hybrid of *Pinguicula* × *Tina* (*Pinguicula agnata* × *Pinguicula zecheri*) purchased from Gartneriet Lammehave (Ringe, Denmark) and *Drosera capensis* in our experiments (Fig. 1A). *Pinguicula* × *Tina* is famous for being vigorous and easy to grow, with many flowers, and producing large leaves with a sufficient amount of digestive fluid for analyses. Plants were grown at the Department of Biophysics of Palacký University in Olomouc, Czech Republic, under standard greenhouse conditions. Plants were grown in plastic pots filled with well-drained peat moss, placed in a tray ﬁlled with distilled water to a depth of 1–2 cm. During the experiments the plants were placed in a growth chamber maintained at 21–22 °C and 100 μmol m^−2^ s^−1^ photosynthetically active radiation, with a 16/8 h light/dark period.

Fruit ﬂies (*Drosophila melanogaster*) were used as a model prey. Flies were cultured from eggs in a carbohydrate-rich medium and were provided by the Department of Genetics, Faculty of Natural Sciences Comenius University in Bratislava, Slovakia. Before the experiments, adult ﬂies were cooled in a refrigerator at 4 °C for 15 minutes to facilitate manipulation. Ten fruit flies were placed on one leaf surface or one flower stalk of plant under study (Fig. 1B, C). After 2 h and 24 h, 10 control and 10 fed leaves were cut off the plant using a scalpel, and leaf blades were submerged one at a time in 4 ml of 50 mM sodium acetate buffer solution (pH 5.0) for 3 min to collect the exudates. Because of seasonal blooming, the limited number and low biomass of flower stalks available were collected only after 24 h.

In the experiments with jasmonates (see below), plants were sprayed with 1 mM jasmonic acid or 100 µM coronatine in 0.001% Tween 20. Control plants were sprayed with 0.001% Tween 20 only. The leaf exudates were collected after 24 h as described above. For this experiment, sundew plants (*D. capensis*) were used as a positive control, as this species is known to increase digestive enzyme synthesis in response to the exogenous application of jasmonates (Krausko *et al.*, 2017). For experiments with hypertonic NaCl solution, 20 µl drops of 5% NaCl or distilled water (as a control) were applied on the glandular leaf surface and collected using a pipette after 15 min. This time point was chosen as sufficiently long to induce the flow of water from the glands but too short for the synthesis and secretion of digestive enzymes *de novo* (based on our experience with Venus flytrap; Jakšová *et al.*, 2020; Pavlovič *et al.*, 2020). For wounding experiments, a leaf was wounded with a needle once for electrical signal measurement or 10–15 times for phytohormone analyses (see below).

### Extracellular recording of electrical signals

Changes in the surface potential were measured by using non-polarizable Ag–AgCl surface electrodes (Scanlab Systems, Prague, Czech Republic) moistened with a drop of conductive EV gel (Hellada, Prague, Czech Republic) that is commonly used in electrocardiography. The electrode was attached on the abaxial side of the leaf either beneath an applied fly (*D. melanogaster*) or 1 cm from a wounding site that was made with a needle. The electrical signals were recorded by a non-invasive device inside a Faraday cage according to Ilík *et al.* (2010). The reference electrode was taped to the side of the plastic pot containing the plant, submerged in 1–2 cm of water in a dish beneath the pot. The electrodes were connected to an amplifier [gain 1–1000, noise 2–3 μV, bandwidth (–3 dB) 10^5^ Hz, response time 10 μs, input impedance 10^12^ Ω]. The signals from the amplifier were transferred to an analogue–digital PC data converter (eight analogue inputs, 12-bit converter, ±10 V, PCA-7228AL, supplied by TEDIA, Plzeň, Czech Republic), collected every 6 ms.

### Measurements of enzyme activities

The proteolytic activity of digestive fluid was determined by incubating 150 µl of the collected sample of digestive fluid with 150 µl of 2% (w/v) bovine serum albumin in 200 mM glycine–HCl (pH 3.0) at 37 °C for 2 h. The reaction was stopped by the addition of 450 µl of 5% (w/v) trichloroacetic acid (TCA). Samples were incubated on ice for 10 min and then centrifuged at 20 000 *g* for 10 min at 4 °C. The amount of released non-TCA-precipitable peptides was used as a measure of proteolytic activity, which was determined by comparing the absorbance of the supernatant at 280 nm with that of a blank sample with a Specord 250 Plus double-beam spectrophotometer (Analytik Jena). One unit of proteolytic activity is defined as an increase of 0.001 min^–1^ in the absorbance at 280 nm (Matušíková *et al.*, 2005).

We used 5 mM 4-nitrophenyl phosphate (Sigma-Aldrich) in 50 mM acetate buffer (pH 5) to estimate the activity of acid phosphatases. A 50 µl sample of the collected digestive ﬂuid was added to 500 µl of the acetate buffer and mixed with 400 µl of the substrate. As a control, 400 µl of substrate solution was added to 550 µl of buffer. Mixed samples were incubated at 25 °C for 2 h. Thereafter, 160 µl of 1.0 M NaOH was added to terminate the reaction. Absorbance was measured at 410 nm with a Specord 250 Plus double-beam spectrophotometer (Analytik Jena). The calibration curve was determined using 4-nitrophenol and the activities were expressed in µmol ml^−1^ h^−1^.

Amylase activity was measured using an amylase assay kit (Sigma-Aldrich). Ethylidene-pNP-G7 was used as a substrate, which upon cleavage by amylase generates 4-nitrophenyl. A 20 µl aliquot of collected digestive fluid was added to a 96-well plate and adjusted to 50 μl with the amylase assay buffer. Then 100 µl of substrate was added, the reaction mixture was incubated at 25 °C, and absorbance at 405 nm was measured every 15 min for 2 h using a SynergyMx microplate reader (BioTek Instruments, Winooski, VT, USA). Positive control (amylase enzyme) and 4-nitrophenyl standard at different concentrations were incubated under the same conditions on the same microplate.

Chitinase activities were measured using a fluorimetric chitinase assay kit (Sigma Aldrich). We used 4-methylumbelliferyl *N*-acetyl-β-d-glucosaminide, 4-methylumbelliferyl *N*,*N*′-diacetyl-β-d-chitobioside, and 4-methylumbelliferyl β-d-*N*,*N*′,*N*″-triacetylchitotriose for the detection of β-*N*-acetylglucosaminidase (exochitinase), chitobiosidase, and endochitinase activities, respectively, according to the manufacturer’s instructions. A 10 μl aliquot of collected digestive fluid was incubated with 90 μl of substrate working solution at 37 °C and after 2 h the reaction was stopped by the addition of 200 μl of sodium carbonate provided in the kit. The fluorescence of liberated 4-methylumbelliferone was measured in alkaline pH using a SynergyMx microplate reader (BioTek Instruments, Winooski, VT, USA) with excitation at 360 nm and emission at 450 nm. Chitinase from *Trichoderma viride* (positive control) and 4-methylumbelliferone standard at different concentrations were incubated under the same conditions on the same microplate.

All enzyme activities were measured on pooled samples from 10 leaves from 3 plants to have sufficiently concentrated samples within the limit of detection.

### SDS-PAGE electrophoresis

Digestive ﬂuid collected for the enzyme assays was subjected to SDS-PAGE. The samples were heated and denatured for 30 min at 70 °C and then mixed with modiﬁed Laemmli sample buffer to a ﬁnal concentration of 50 mM Tris–HCl (pH 6.8), 2% SDS, 10% glycerol, 1% β-mercaptoethanol, 12.5 mM EDTA, and 0.02% bromophenol blue. The same volume of digestive ﬂuid was electrophoresed in 10% (v/v) SDS-polyacrylamide gel (Schägger, 2006). The proteins in the gels were visualized by silver staining (ProteoSilver; Sigma Aldrich).

### Proteomic analysis of digestive fluid

Freshly collected digested fluid from fed plants was divided into 1 ml aliquots, which were subsequently frozen in liquid nitrogen and lyophilized overnight. The dry residue corresponding to one aliquot was adjusted to 100 µl with 10× cOmplete™ Protease Inhibitor Cocktail (Roche, Switzerland) in 100 mM NaCl, and proteins were precipitated using the TCA/acetone method. Briefly, the protein sample was thoroughly mixed with 8 volumes of ice-cold acetone and 1 volume of TCA, and the resulting solution was kept at –20 °C for 1 h. The protein pellet was recovered by centrifugation at 20 000 g and 4 °C for 10 min, rinsed twice with 2 volumes of ice-cold acetone (Kim *et al.*, 2006), dissolved in Laemmli sample buffer, and separated by SDS-PAGE (Laemmli, 1970). The resolved proteins were stained with colloidal Coomassie (Candiano *et al.*, 2004) and digested in-gel with raffinose-modified trypsin (Šebela *et al.*, 2006) as described elsewhere (Shevchenko *et al.*, 2006). Peptides were cleaned on home-made C18 StageTips (Rappsilber *et al.*, 2008), and mass spectrometry (MS) analysis was done on a UHR-QTOF maXis tandem mass spectrometer (Bruker Daltonik, Bremen, Germany) coupled to a RSLCnano nanoflow capillary liquid chromatography system (Dionex, Thermo Fisher Scientific, Sunnyvale, CA, USA) via online nanoESI source (Bruker Daltonik, Bremen, Germany). The specific settings of the chromatography system and the mass analyzer were identical to those described previously (Simerský *et al.*, 2017).

The acquired MS data were either processed by classical MASCOT searches against a selected database or subjected to *de novo* sequencing. In the first case, the precursor and fragmentation data were extracted from raw data using DataAnalysis v 4.3 x64 (Bruker Daltonik, Bremen, Germany), exported into MGF files, and uploaded to Protein Scape v. 2.1 (Bruker Daltonik, Bremen, Germany). Peptide and protein searches were performed employing the MASCOT algorithm (v2.2.07, in-house server; Matrix Science, London, UK) against an order Lamiales-specific protein database (NCBI; 325 526 sequences; downloaded 23 October 2017) that was supplemented with common protein contaminants. The following parameters were used for each MASCOT search: MS and MS/MS tolerance were set at ±25 ppm and ±0.03 Da, respectively; trypsin was selected as the protease and two missed cleavages were allowed; carbamidomethylation of cysteine was included as a fixed modification; and *N*-terminal protein acetylation and methionine oxidation were selected as variable modifications. A positively identified protein had to fulfil the following parameters: contain at least one peptide with identity score calculated by the MASCOT algorithm (a cut-off score required for the other assigned peptides was 25 with *P*-value of 0.05); pass over a protein cut-off score of 30. For *de novo* sequencing, the DeNovoGUI interface (v1.16.0; Muth *et al.*, 2014) containing the Novor (Ma, 2015), DirecTag (Tabb *et al.*, 2008), PepNovo (Frank and Pevzner, 2005), and pNovo (Chi *et al.*, 2010) algorithms was applied to generate full-length peptide sequences directly from raw data. The same settings were adopted as for the MASCOT searches described above. To assign all obtained *de novo* peptide sequences, a local pBLAST search was carried out against a compiled list of proteins identified in digestive fluids from all carnivorous plant species that have been examined to date. The BLAST hits were filtered by similarity with following requirements: an alignment length of at least five amino acids; cut-off values for overall identity and positivity were set at 75%. After manual quality control of peptide spectra, all assigned *de novo* peptides were considered as positive identifications.

### Quantification of phytohormones

At 2 h and 24 h after prey feeding or wounding with a needle, leaves were collected from control and fed plants and immediately (within 10 s) frozen in liquid nitrogen and stored at –80 °C until analysis. Quantification of jasmonic acid (JA), JA-Ile, *cis*-12-oxo-phytodienoic acid (*cis*-OPDA), abscisic acid (ABA), salicylic acid (SA), and indole-3-acetic acid (IAA) was performed according to the modified method described by Floková *et al.* (2014). Briefly, frozen plant material (20 mg) was homogenized and extracted using 1 ml of ice-cold 10% methanol/H_2_O (v/v). A cocktail of stable isotope-labeled standards was added as follows: 10 pmol of [^2^H_6_]JA, [^2^H_2_]JA-Ile, [^2^H_5_]OPDA, [^2^H_6_]ABA, and [^13^C_6_]-IAA, and 20 pmol of [^2^H_4_]SA (all from Olchemim Ltd, Czech Republic) per sample to validate the LC-MS/MS method. The extracts were purified using Oasis^®^ HLB columns (30 mg 1 ml^–1^, Waters) and hormones were eluted with 80% methanol. The eluent was evaporated to dryness under a stream of nitrogen. Phytohormone levels were determined by ultra-high performance liquid chromatography-electrospray tandem mass spectrometry (UPLC-MS/MS) using an Acquity UPLC^®^ I-Class System (Waters, Milford, MA, USA) equipped with an Acquity UPLC CSH^®^ C_18_ column (100 × 2.1 mm; 1.7 µm; Waters) coupled to a Xevo^™^ TQ-S MS triple quadrupole mass spectrometer equipped with electrospray ionization technique (Waters MS Technologies, Manchester, UK). Three independent technical measurements were performed on 5–15 biological replicates.

### Statistical analyses

Throughout this paper, data are presented as means ±SD. To evaluate the signiﬁcance of differences between the control and treated plants, two-tailed Student’s *t*-tests was used (Origin 2015, Northampton, MA, USA). Before the statistical tests, the data were analyzed for normality and homogeneity of variance. When non-homogeneity was present, the *t*-test was used with the appropriate corrected degrees of freedom (Welch’s *t*-test).

## Results

### Electrical signaling

**Fig. 2.**
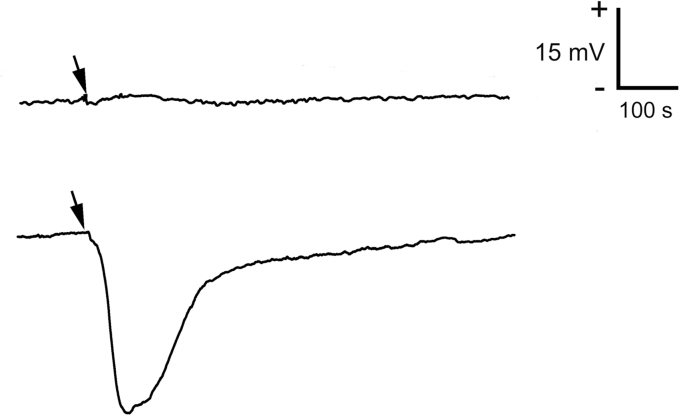
Extracellular membrane potential in response to prey (*Drosophila melanogaster*) applied on the trap surface (upper trace) and wounding (lower trace) in *Pinguicula* × *Tina*. Arrows indicate the time point of stimulus application. These representative records are shown from five independent measurements.

The presence of live prey did not elicit any electrical signal in the leaf for the duration of measurements (6 h). By contrast, wounding with a needle elicited depolarization of membrane potential, a typical variation potential (VP) with amplitude 15–50 mV and duration 200–400 s (negative voltage shift recorded extracellularly, representing intracellular depolarization; Fig. 2).

### Insect prey-induced enzyme activity

**Fig. 3.**
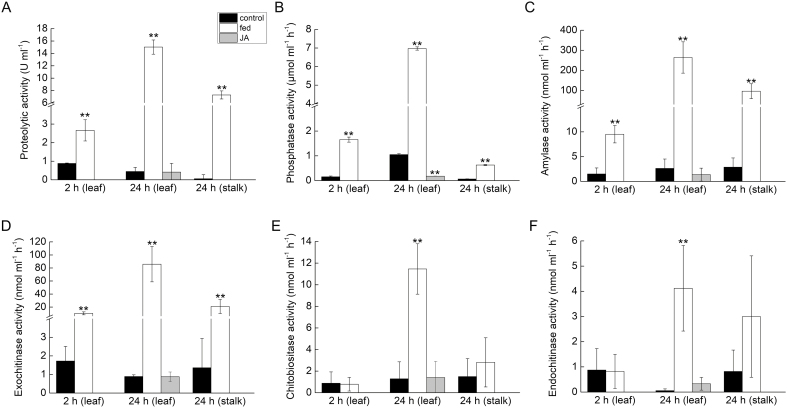
Enzyme activities in *Pinguicula* × *Tina* in response to insect prey feeding and jasmonic acid (JA) application. Enzyme activities were measured 2 h and 24 h after feeding in leaf exudates, 24 h after feeding in flower stalk exudates, and 24 h after the application of 1 mM JA on the trap surface. (A) Proteolytic activity. (B) Phosphatase activity. (C) Amylase activity. (D) Exochitinase activity. (E) Chitobiosidase activity. (F) Endochitinase activity. Data are mean ±SD (*n*=5–6). Significant differences between control and fed plants, and between control and JA-applied plants, were evaluated by Student’s *t*-test: *P<0.05, **P<0.01.

The activities of proteases, acid phosphatases, amylases, and exochitinases increased 2 h after prey feeding (Fig. 3A–D). After 24 h, all measured enzyme activities in leaf exudates were significantly increased (Fig. 3A–F). Surprisingly, the flower stalk exudates also had increased proteolytic, acid phosphatase, amylase, and exochitinase activities 24 h after feeding (Fig. 3A–D).

### Composition of digestive fluid

Our homology based-identification strategy enabled the identification of 14 protein sequences covering 8 different catalytic activities, which included aspartic and cysteine proteases, peroxidases, esterase/lipase and endonuclease. Interestingly, the presence of alpha-amylase, an enzyme that has not been described before in the digestive fluids of the other carnivorous plants, was identified as well (Table 1; see also Supplementary Table S1 and Fig. S1 at *JXB* online).

**Table 1.** Proteins identified by mass spectrometry analysis in *Pinguicula* × *Tina* digestive fluid collected 24 h after feeding on fruit flies

MS data processing method	Identification characteristics				
**MASCOT search**	**Detected sequence**	**Assigned protein**	**Accession** ^***a***^	**MASCOT score**	**Peptides/PSMs/SC** ^***b***^
	LAASILR	Peroxidase 10-like	KZV23101.1	33.5	1/3/2.1
	AVADIVINHR	Alpha-amylase	EPS60632.1	38.9	1/1/2.8
	GILQAAVQGELWR	Alpha-amylase	KZV28895.1	39.9	1/4/3.4
***De novo* sequencing pBLAST search**	**Detected sequence**	**Homologous protein**	**Accession** ^***a***^	***De novo* score**	**pBLAST identity/** **positivity** ^***c***^
	TVPMVLNGAGLLNMGPPHMK	Nepenthesin II	BAD07475.1	30.28	77/88
	WESSLNWVLCMK	Asp protease	GAV80475.1	30.93	75/75
	HQMLVALQYYCNR	Cysteine protease	BAW35427.1	32.63	83/83
	MVQGGSGKVAQQTLAAN	Desiccation-related protein	BAW35440.1	31.03	75/100
	GRLMVAGLGGLGMKER PNKFGVGLGGLGLMQR	Cinnamyl alcohol dehydrogenase	-	35.07^*d*^ 35.08^*d*^	87/87^*d*^ 100/100^*d*^
	MPVDFNVTATFHLQ	Leu-rich repeat protein NrLRR1	-	33.77	75/100
	SLNLNSLRGNVK	Peroxidase	BAM28609.1	32.28	100/100
	YYFNLNYPEGFTK	Beta-xylosidase	AAX92967.1	40.02	85/85
	TLLSDLVNSTTAMMK	Peroxidase	-	34.23	77/100
	ARMTNMRNKVQQVQQNMPR	GDSL esterase/lipase	XP_004232991.1	30.64	77/77
	AQKRNWVQQWQR	Endonuclease 2	-	32.59	100/100

^*a*^ NCBI database accession. ^*b*^ PSMs, peptide-spectrum matches; SC, sequence coverage in %.  ^*c*^ pBLAST identity and positivity in %.  ^*d*^ Characteristics for two independent peptide hits acquired for the respective protein.

### Jasmonates are not responsible for induction of enzyme activity

**Fig. 4.**
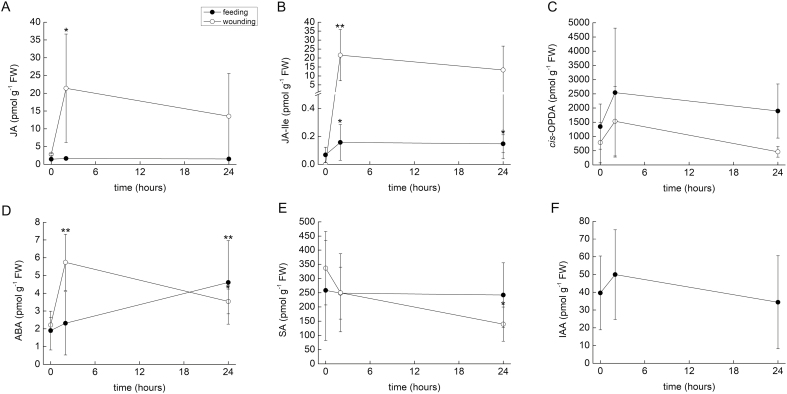
Tissue levels of phytohormones in *Pinguicula* × *Tina* in response to feeding and wounding. (A) Jasmonic acid (JA). (B) Isoleucine conjugate of jasmonic acid (JA-Ile). (C) *cis*-12-oxophytodienoic acid (*cis*-OPDA). (D) Abscisic acid (ABA). (E) Salicylic acid (SA). (F) Indole-3-acetic acid (IAA). Data are mean ±SD (*n*=5–15). Significant differences between control (time 0) and fed plants after 2 h and 24 h were evaluated by Student’s *t*-test: *P<0.05, **P<0.01.

The level of JA did not significantly increase at 2 h or 24 h after feeding plants with fruit flies. To investigate whether *Pinguicula* increases JA in response to damaging stimuli, leaves were wounded 10–15 times with a needle. Wounding resulted in a 10-fold increase of JA after 2 h; this increase was significant (Fig. 4A). While feeding resulted in a slight (1-fold) but significant increase of JA-Ile, wounding caused a 200-fold increase (Fig. 4B). The level of the JA precursor *cis*-OPDA was not altered significantly (Fig. 4C). Wounding also increased the level of ABA after 2 h and 24 h, whereas feeding increased the level of ABA only after 24 h (Fig. 4D). The level of SA did not increase in response to either stimulus, and only a significant decrease in response to wounding was detected at 24 h (Fig. 4E). The level of IAA level remained more or less constant in fed plants (Fig. 4F); IAA analysis in wounded leaves failed.

**Fig. 5.**
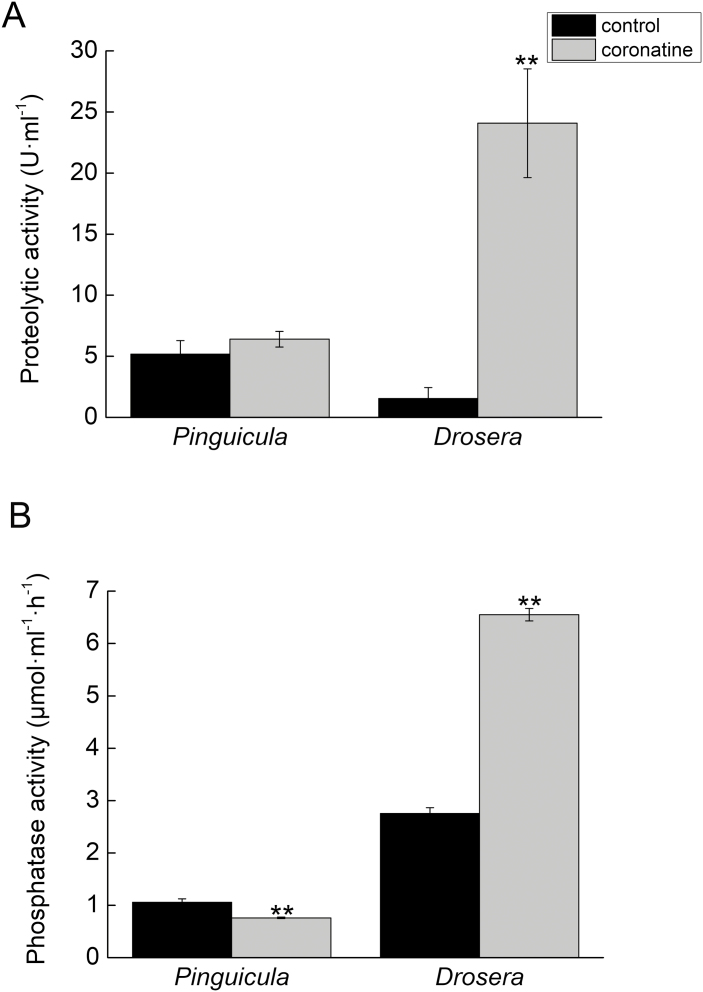
Effect of application of 100 µM coronatine on enzyme activities in leaf exudates of *Pinguicula* × *Tina* and *Drosera capensis*. (A) Protease activity. (B) Phosphatase activity. Data are mean ±SD (*n*=5). Significant differences between control and coronatine-treated plants were evaluated by Student’s *t*-test: *P<0.05, **P<0.01.

**Fig. 6.**
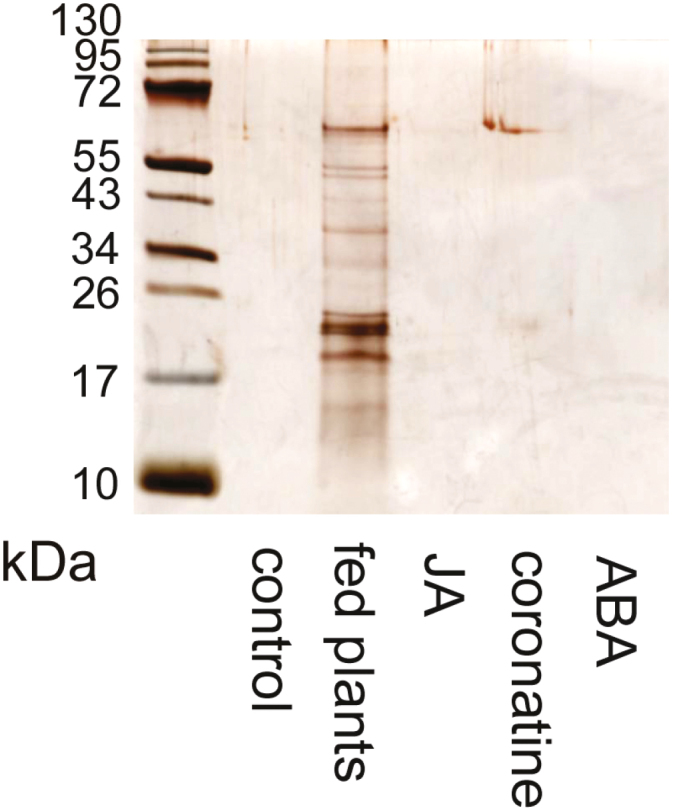
Silver-stained SDS-PAGE of digestive ﬂuid released in response to different stimuli in *Pinguicula* × *Tina*. The same volume of digestive fluid 24 h after different treatments was electrophoresed and the proteins were separated in 10% (v/v) SDS-polyacrylamide gel and silver stained. ABA, abscisic acid; JA, jasmonic acid.

To confirm that jasmonates are not involved in enzyme secretion, we applied JA and coronatine (a molecular mimic of JA-Ile) exogenously. Neither JA nor coronatine was able to induce enzyme activity (Figs 3 and 5). SDS-PAGE confirmed that JA, ABA, and coronatine did not induce the secretion of proteins, and the protein profile of treated plants was comparable to that of control plants (Fig. 6). For control experiments, we used sundew plants (*D. capensis*), which are known to regulate enzyme production through jasmonates (Krausko *et al.*, 2017). Sundew plants showed a significant increase in enzyme activity in leaf exudates after coronatine treatment (Fig. 5). In response to exogenous coronatine, sundew plants folded their tentacles and traps not only locally to the site of coronatine application but also systemically, and both local and systemic leaves started to secrete digestive fluid. No such behaviour was observed in *Pinguicula* × *Tina* plants, which cannot fold their leaves.

### Rapid efflux of water is responsible for induction of phosphatase activity

**Fig. 7.**
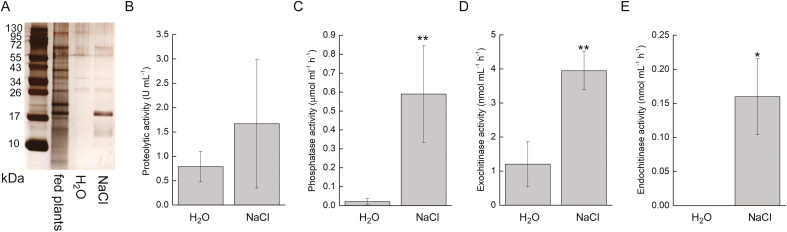
Silver-stained SDS-PAGE and enzyme activities in the digestive ﬂuid released in response to salt application in *Pinguicula* × *Tina*. (A) Protein profile resolved by SDS-PAGE. The same volume of digestive fluids 15 minutes after H_2_O and 5% NaCl application and 24 h after feeding was electrophoresed and the proteins were separated in 10% (v/v) SDS-polyacrylamide gel and silver stained. (B) Proteolytic activity. (C) Phosphatase activity. (D) Exochitinase activity. (E) Endochitinase activity. Data are mean ±SD (*n*=3–6). Significant differences between H_2_O and 5% NaCl treated plants were evaluated by Student’s *t*-test: *P<0.05, **P<0.01

To induce a rapid efflux of water from the digestive glands of *Pinguicula* plants, we applied hypertonic 5% NaCl solution to the glandular leaf surface. The secretion was collected 15 min later for further analyses. Measurements of enzyme activity showed a 30-fold increase of phosphatase activity in digestive fluid within 15 min (Fig. 7B). The proteolytic activity was not significantly increased in comparison to controls (leaves treated with water) (Fig. 7C). Amylase and chitobiosidase activities were not detected. Exochitinase and endochitinase activities were slightly but significantly increased relative to controls (Fig. 7D, E). SDS-PAGE showed the clear appearance of some proteins in the digestive fluid of NaCl-treated leaves. The most prominent was the appearance of a band at ~20 kDa (Fig. 7A).

## Discussion

In order to save available resources in nutrient-poor environments, carnivorous plants produce digestive enzymes not constitutively but in response to prey capture. Mechanical and chemical stimuli from insect prey have an indispensable role in this process (for review, see Pavlovič and Mithöfer, 2019). The carnivorous plants with active trapping mechanisms (e.g. *Drosera* and *Dionaea* spp.) rely on both stimuli (Libiaková *et al.*, 2014; Bemm *et al.*, 2016; Krausko *et al.*, 2017; Jakšová *et al.*, 2020). The carnivorous plants with passive trapping mechanisms (*Nepenthes* and *Sarracenia* spp.) rely solely on chemical stimuli (Gallie and Chang, 1997; Yilamujiang *et al.*, 2016; Saganová *et al.*, 2018). The presence of chitin, protein, or different ions (e.g. NH_4_^+^) has been shown to be effective in induction processes (Libiaková *et al.*, 2014; Yilamujiang *et al.*, 2016; Saganová *et al.*, 2018; Jakšová *et al.*, 2020). All these stimuli increase the level of jasmonates, which transcriptionally activate the genes encoding digestive enzymes (Bemm *et al.*, 2016; Yilamujiang *et al.*, 2016; Pavlovič *et al.*, 2017; Jakšová *et al.*, 2020). However, all these studies were confined to carnivorous plants within the order Caryophyllales.

In this study, we showed that the butterwort (*Pinguicula* × *Tina,* order Lamiales) had increased enzyme activities in digestive fluid from leaves in response to prey capture. Interestingly, the enzyme activities were also increased in flower stalk exudate, which indicates that the flower stalk of *Pinguicula* is an additional carnivorous organ—a unique adaptation among carnivorous plants. This is consistent with the uptake of nitrogen from prey captured by the flower stalk, as was previously documented in *Pinguicula vulgaris* and *Pinguicula villosa* (Hanslin and Karlsson, 1996). After prey capture, MS revealed the presence of enzymes in leaf exudate well known from other non-related genera of carnivorous plants, such as cysteine and aspartic proteases, endonuclease, and peroxidase (Hatano and Hamada, 2008, 2012; Schulze *et al.*, 2012; Lee *et al.*, 2016; Rottloff *et al.*, 2016; Fukushima *et al.*, 2017; Krausko *et al.*, 2017). We propose the names ‘pinguiculain’ for cysteine protease and ‘pinguiculasin’ for aspartic protease, following the nomenclature of Takahashi et al. (2009, 2012). This finding supports the hypothesis that carnivorous plants with independent origins repeatedly co-opted the same plant defense protein lineages to acquire digestive physiology (Fukushima *et al.*, 2017). However, we also found one unique enzyme that has not been identified in the secretome of carnivorous plants before: alpha-amylase. Amylase is an enzyme that catalyzes the hydrolysis of starch, a polysaccharide produced by most green plants as an energy store. Our finding is consistent with the work of Heslop-Harrison and Knox (1971), which demonstrated amylase activity in the digestive glands of *Pinguicula*. The flat leaves of *Pinguicula* often trap significant amounts of plant material (Darwin, 1875) and amylases may help to digest this alternative source of carbon, implying that *Pinguicula* is a true mixotroph. When grown in axenic culture, plants of *Pinguicula lusitanica* showed significant increases in the numbers of leaves and flowers when fed with pine pollen (Harder and Zemlin, 1968). This ‘vegetarianism’ of carnivorous plants within the order Lamiales is not rare; on the contrary, it is very common in the genus *Utricularia* (Peroutka *et al.*, 2008, Koller-Peroutka *et al.*, 2015) and probably also in *Genlisea* (Plachno and Wolowski, 2008). The presence of alpha-amylase in digestive fluid might represent another example of non-defense-related genes that have been co-opted for the syndrome of carnivory. The class V β-1,3-glucanases in *Nepenthes* and *Dionaea*, like alpha-amylase, are involved in embryo and pollen development and germination rather than defense responses (Michalko *et al.*, 2017). The detection of phosphatase and chitinase activities (Fig. 3) but the absence of corresponding enzymes in the acquired dataset, together with rather modest coverages obtained for the assigned sequences, indicate that our proteomic analysis was not exhaustive and more enzymes could be present in the digestive fluid of *Pinguicula*. The most probable cause of this disparity is the lack of appropriate genomic background, which is a prerequisite for each protein identification experiment.

If these proteins were repeatedly co-opted for digestive physiology, it is tempting to assume that the signaling pathway would also be. Jasmonate signaling has been investigated in only three genera of carnivorous plants so far (*Dionaea*, *Drosera*, and *Nepenthes*), all of which are within the order Caryophyllales. All of these carnivorous plants increase their endogenous level of jasmonates (JA and JA-Ile) in response to prey capture and secrete enzymes in response to the exogenous application of these jasmonates (Nakamura *et al.*, 2013; Yilamujiang *et al.*, 2016; Krausko *et al.*, 2017; Pavlovič *et al.*, 2017). However, in this study we showed that neither of these phenomena occur in *Pinguicula*. A slight increase in JA-Ile was found in response to feeding (Fig. 4B), but such an increase could be caused by the presence of chitin in the applied insect exoskeleton and has probably no function in plant carnivory. This is supported by the fact that the exogenous application of JA and coronatine could not mimic insect prey and enzyme production was not increased in response to their application (Figs 3 and 5). The slight increase of endogenous ABA after feeding (Fig. 4B) was probably also caused by the presence of chitin in the insect exoskeleton, which may increase ABA concentrations (Iriti and Faoro, 2008; Iriti *et al.*, 2009, Jakšová *et al.*, 2020). The SDS-PAGE protein profiles in response to coronatine, JA, and ABA application strongly differ from those induced by insect prey and resemble control (unfed) plants, with no detectable protein bands (Fig. 6). In carnivorous plants of the genera *Drosera* and *Dionaea*, the exogenous application of jasmonates induces the same protein spectra as the application of live prey (compare Fig.7 in Krausko *et al.*, 2017, or Fig. 6 in Pavlovič *et al.*, 2017). In other words, the application of jasmonates to *Pinguicula* cannot mimic the presence of insect prey, which is in contrast to the response to exogenous jasmonates of carnivorous plants in the order Caryophyllales. To investigate whether other plant hormones might activate enzyme secretion by *Pinguicula*, we applied 100 µM gibberellic acid (GA_3_), 250 µM SA, and 200 nM IAA, but none of these phytohormones was able to trigger significant enzyme activities in digestive fluid (data not shown).

The absence of jasmonate signaling in the genus *Pinguicula* could be explained by the absence of electrical signaling in its prey-detection system, in contrast to *Drosera* and *Dionaea* (Böhm *et al.*, 2016; Krausko *et al.*, 2017, Pavlovič *et al.*, 2017). In these genera, electrical and jasmonate signaling enable the coordinated activation of digestive processes in neighbouring glands that have not touched the insect prey. In *Pinguicula*, the activation of digestive glands is confined to the release of digestive fluid in response to chemical stimuli from the prey itself and the glands that have made contact with it (Heslop-Harrison and Knox, 1971). However, in some carnivorous plant species the presence of chemical signals from insect prey alone is able to trigger JA accumulation, as was documented in passive pitcher traps of *Nepenthes* (Yilamujiang *et al.*, 2016). This indicates that rapid electrical signals are not necessary for the induction of JA signaling in carnivorous plants. Thus, their absence in prey detection cannot account for the observation that *Pinguicula* does not use jasmonate signaling for the regulation of enzyme secretion.

The mechanism triggering enzyme secretion in the genus *Pinguicula* remains a subject for future research, given that our findings indicate that the jasmonates were not co-opted for plant carnivory in this genus. Heslop-Harrison and Knox (1971) proposed that the enzymes are pre-synthesized and stored in the vacuoles and cell walls of digestive glands, and are released only by the flux of water triggered by prey capture. They suggested total autolysis of the cells, in contrast to Vassilyev and Muravnik (1988a, b), who argued that secretory cells of the digestive glands remain highly active during the entire period of prey digestion. Vassilyev and Muravnik (1988a, b) also suggested that additional digestive enzymes are synthesized *de novo* after stimulation, as occurs in Venus flytrap. Based on our experiments with NaCl, it seems that at least phosphatases are pre-synthesized and are only flushed away from the cell walls of the digestive glands by chloride ion movement (Heslop-Harrison and Heslop-Harrison, 1980). Indeed, a cytochemical study of the leaf gland enzymes in *Pinguicula* showed the presence of phosphatases in the spongy radial walls of the head of the unstimulated gland, which are clearly flushed away by the flux of water (Heslop-Harrison and Knox, 1971). However, some of the enzymes need to be synthesized *de novo* (e.g. proteases and amylases). The signal that triggers the expression of these enzymes remains unknown.

## Conclusions

Our study clearly shows that whereas the proteomic composition of digestive fluid is similar among different orders of carnivorous plants (with the exception of alpha-amylase), the mode of their regulation may differ. This finding is consistent with the study of Nishimura *et al.* (2013), who found S-like RNases in three genera of carnivorous plants from two orders, but with different regulation of their expression. Although the genus *Pinguicula* shows strongly enhanced enzyme secretion in response to prey capture, jasmonates are not involved in this process. The hypothesis that the digestive enzymes are pre-synthesized and only flushed away by water outflow cannot be accepted entirely. The type of signal that is involved after prey capture in *Pinguicula* remains unknown.

## Supplementary data

Supplementary data are available at *JXB* online.

Fig. S1. Protein profile of the digestive fluid from *Pinguicula* × *Tina* in response to feeding.

Table S1. Proteins identified in the digestive fluid of *Pinguicula* × *Tina.*

eraa159_suppl_Supplementary_table

eraa159_suppl_Supplementary_figure
