# Supplementary material for: Jasmonate-independent regulation of digestive enzyme activity in the carnivorous butterwort Pinguicula × Tina
Source: J Exp Bot. 2020 Mar 27;71(12):3749–58. doi: 10.1093/jxb/eraa159 (PMC7307851; doi:10.1093/jxb/eraa159)
Supplement: eraa159_suppl_Supplementary_figure [file eraa159_suppl_supplementary_figure.pdf]

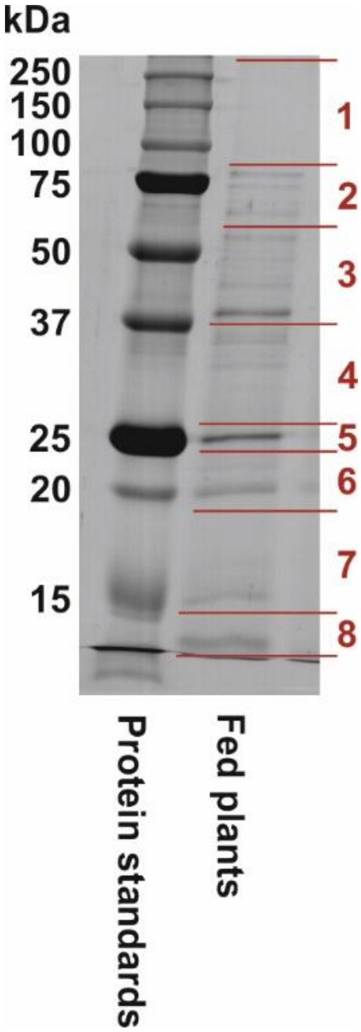

Fig. S1 Protein profile of the digestive fluid from *Pinguicula* x *Tina* in response to feeding. An aliquot (1 mL) of freshly collected digestive fluid secreted in response to feeding was frozen in liquid nitrogen and lyophilized overnight. The resulting dry residue was taken to 100 μL with 100mM NaCl, 10x cOmplete protease inhibitor cocktail (Roche, Switzerland) and proteins were precipitated using the TCA/acetone method. The obtained protein pellet was dissolved in Laemmli sample buffer, separated by SDS-PAGE and stained with colloidal Coomassie. Then, the gel was divided into 8 slices (shown with numbers) and the resolved proteins were digested in-gel with trypsin with raffinose-modified trypsin as described elsewhere. The resultant tryptic digest was cleaned on home-made C18 StageTips and analyzed by LC-ESI-MS/MS according to Simerský et al. (2017). For more detailed information, see Materials and Methods and the Supplementary Table 1.
